# The Relationship Between Hemodynamic Responses During Head-Up Tilt Testing and Parameters of Infection in Post-COVID Syndrome, Chronic Fatigue Syndrome, and Late-Stage Lyme Disease

**DOI:** 10.3390/v17111430

**Published:** 2025-10-28

**Authors:** Branislav Milovanovic, Nikola Markovic, Masa Petrovic, Smiljana Stojanovic, Vasko Zugic, Milijana Ostojic, Milovan Bojic

**Affiliations:** 1 Institute for Cardiovascular Diseases “Dedinje”, 11000 Belgrade, Serbiastojanovicsmiljana@gmail.com (S.S.);; 2 Faculty of Medicine, University of Belgrade, 11000 Belgrade, Serbia

**Keywords:** neurocardiology, late-stage Lyme disease, head-up tilt test, chronic fatigue syndrome, post-COVID

## Abstract

Autonomic nervous system (ANS) dysfunction has emerged as a central feature of post-infectious syndromes, including post-COVID syndrome (PCS), chronic fatigue syndrome (CFS), and late-stage Lyme disease. This cross-sectional study included 1036 patients evaluated in the Neurocardiological Laboratory of the Institute for Cardiovascular Diseases “Dedinje,” divided into four groups: PCS, CFS after COVID-19, CFS of insidious onset, and Lyme disease. All patients underwent head-up tilt testing (HUTT), and serological testing was performed in accredited institutions. The Lyme disease group demonstrated the highest prevalence of positive HUTT responses and a significantly greater frequency of orthostatic hypotension and syncope. Approximately 50–65% of patients in the PCS and Lyme groups were positive for IgM antibodies against at least one microorganism, with more than 10% showing positivity for three or more pathogens. Logistic regression analysis revealed that, beyond classical hemodynamic parameters, antibody status served as a significant predictor of HUTT outcomes, with specific associations identified for HSV-1, HHV-6, *Coxiella burnetii*, *Toxoplasma gondii*, and *Borrelia* spp. Multinomial regression further indicated that negative IgG antibodies, particularly to HSV-1 and VZV, predicted Lyme disease group membership. These findings support the hypothesis that ANS dysfunction in post-infectious syndromes may be driven by persistent or prior infections, highlighting the need for integrative diagnostic approaches.

## 1. Introduction

The autonomic nervous system (ANS) is composed of numerous neurons and pathways whose primary function is the unconscious regulation of non-motor end organs, thereby maintaining homeostasis across the organism [1]. Although the ANS operates largely at an unconscious level, its functions are highly integrated with other neural networks, and the boundaries separating autonomic from non-autonomic subgroups are not strictly defined [1].

Several diagnostic modalities are currently available for assessing ANS function. Objective methods include the head-up tilt test (HUTT), cardiovascular reflex tests described by Ewing (CART), QASAT1 methodology, and heart rate variability (HRV). In contrast, subjective methods include COMPAS-31 and SAS-3 [2,3,4,5,6,7,8]. Impairment of ANS function, commonly referred to as dysautonomia, manifests through a wide range of symptoms, such as postural orthostatic tachycardia syndrome (POTS), orthostatic hypotension (OH), orthostatic intolerance, chronic fatigue, and cognitive impairment [9,10,11,12].

These polymorphic complaints (chronic fatigue, “brain fog,” and orthostatic intolerance) gained considerable attention after the emergence of post-COVID syndrome (PCS), due to the large number of affected patients. The precise pathophysiology of ANS dysfunction following SARS-CoV-2 infection remains unclear. However, several mechanisms have been proposed, including direct viral effects, systemic hyperinflammatory states, cytokine- and hypoxia-induced injury, and small fiber neuropathies [13,14]. Chronic fatigue syndrome (CFS), which is similarly characterized by ANS dysfunction [11,15], presents with nearly identical symptoms. Jason et al. demonstrated that 58% of 465 patients with PCS met the diagnostic criteria for myalgic encephalomyelitis/chronic fatigue syndrome (ME/CFS) [16]. Moreover, approximately two-thirds of patients with CFS report disease onset following “flu-like” symptoms, and the condition has been associated with infectious agents such as cytomegalovirus (CMV), human herpesvirus 6 (HHV-6), parvovirus B19, *Chlamydia pneumoniae*, *Coxiella burnetii*, and adenovirus, among others [17,18].

Similarly, late-stage Lyme disease produces almost identical clinical manifestations. Lyme disease is the most common vector-borne infection in the United States and Europe. Chronic manifestations often develop due to delayed diagnosis, inadequate antibiotic therapy, or pathogen-specific characteristics of *Borrelia* spp., such as high genetic variability, antigenic diversity, and mechanisms of immune evasion and modulation [19]. Adler et al. proposed that chronic inflammation, infection-induced autoimmunity, and neuronal and glial cell death during the acute phase contribute to the development of post-treatment Lyme disease syndrome (PTLDS), while also emphasizing the potential role of dysautonomia as a key, often overlooked, component [20]. Noyes and Kluger further reported case studies in which Lyme disease preceded the onset of POTS [21]. Importantly, *Borrelia* infections are frequently accompanied by co-infections, including *Ehrlichia chaffeensis*, *Bartonella*, *Coxiella*, *Babesia*, *Francisella tularensis* (tularemia), *Rickettsia*, *Mycoplasma pneumoniae*, *Chlamydia pneumoniae*, and parvovirus B19, among others [22].

The aims of this study were to (i) evaluate hemodynamic changes during HUTT in patients with PCS, CFS, and late-stage Lyme disease; (ii) assess the potential for predicting HUTT responses; and (iii) analyze the results of serological testing in these patient groups.

## 2. Materials and Methods

### 2.1. Study Protocol

This cross-sectional study included 1036 patients aged 18 years or older who were examined in the Neurocardiological Laboratory of the Cardiology Clinic at the Institute for Cardiovascular Diseases “Dedinje.” Patients were divided into four groups. The first group, referred to as the post-COVID syndrome (PCS) group, consisted of 97 patients who fulfilled the inclusion criterion of persistent symptoms lasting four weeks or longer after acute SARS-CoV-2 infection [23,24]. The second group included 285 patients diagnosed with chronic fatigue syndrome (CFS) that developed after SARS-CoV-2 infection, representing CFS secondary to PCS. The third group comprised 499 patients diagnosed with CFS of non-COVID-19 origin, based on the 2021 National Institute for Health and Care Excellence (NICE) criteria [25]. The fourth group, designated as the Lyme disease group, consisted of 157 patients presenting with polymorphic symptoms and neurological, cardiological, rheumatological, and systemic manifestations such as chronic fatigue, orthostatic intolerance, cognitive impairment, and migraines, persisting longer than six months, together with a positive two-tier serological protocol for Lyme disease. The two-tier testing required both a positive ELISA/EIA and a positive Western blot for the same class of antibodies (IgM or IgG).

Exclusion criteria applied to all groups with the presence of chronic diseases such as neurological, endocrinological, or autoimmune disorders that could explain the symptoms, as well as neurological diseases known to cause primary autonomic failure, including pure autonomic failure (PAF), multiple system atrophy (MSA), and Parkinson’s disease (PD). In the first three groups, all patients underwent head-up tilt testing (HUTT) using the Westminster protocol (n = 879), while in the Lyme disease group, 112 patients (71.3%) were tested. Complete serological results were available for 29 patients in group 1, 95 patients in group 2, 142 patients in group 3, and all 157 patients in group 4. All serology tests were performed by accredited reference institutions in the Republic of Serbia.

The study was conducted in accordance with the Declaration of Helsinki and approved by the Institutional Ethics Committee of the Institute for Cardiovascular Diseases “Dedinje” (protocol code 6472; approval date 11 December 2024). This work was supported by the Ministry of Education, Science, and Technological Development of the Republic of Serbia (grant 451-03-68/2020-14/200156) and by the Science Fund of the Republic of Serbia (COVANSA grant).

### 2.2. Head-Up Tilt Test (HUTT)

The Westminster protocol was used for HUTT [2]. Prior to testing, each patient spent ten minutes in the supine position. The tilt angle was set at 70 degrees, and the maximum planned test duration was 30 min. The test was considered positive if syncope or severe presyncope occurred, accompanied by a drop in blood pressure or the appearance of bradycardia. Blood pressure and a 12-lead ECG were continuously monitored.

In addition to positive or negative outcomes, several hemodynamic responses were recorded during the test. Extreme variation of systolic blood pressure was defined as a sustained fluctuation greater than 20 mmHg between the maximum and minimum values in either the supine or passive phases, while small variation was defined as a fluctuation between 10 and 20 mmHg. A hypertensive reaction was defined as a sustained blood pressure exceeding 130/90 mmHg during the passive phase, whereas an extreme hypertensive reaction was defined as a sustained blood pressure exceeding 170/120 mmHg. Postural orthostatic tachycardia syndrome (POTS) was defined as a rapid increase in heart rate of more than 30 beats per minute or a heart rate exceeding 120 beats per minute within ten minutes of assuming the upright position in the absence of orthostatic hypotension but with accompanying symptoms of orthostatic intolerance [26]. Orthostatic hypotension (OH) was defined as a sustained decrease in systolic blood pressure greater than 20 mmHg, a decrease in diastolic pressure greater than 10 mmHg, or an absolute systolic pressure lower than 90 mmHg [27]. Bradycardia in the supine position was defined as a heart rate below 60 beats per minute, tachycardia as a heart rate above 100 beats per minute, hypotension as a blood pressure equal to or lower than 90/60 mmHg, and hypertension as a blood pressure exceeding 130/90 mmHg.

### 2.3. Serology Tests

All serological analyses were performed in reference institutions in Serbia, which selected appropriate assays for each microorganism. Blood samples were collected three to five days after the patients’ evaluation in the Neurocardiological Laboratory. Results were reported as either positive or negative. None of the patients displayed acute infectious symptoms such as fever, sore throat, dyspnea, nasal congestion, chills, or sweats at the time of testing.

The panel of serological tests included viral agents (HSV-1, HSV-2, varicella-zoster virus, cytomegalovirus, Epstein–Barr virus, HHV-6, adenovirus, parvovirus B19, coxsackievirus, and SARS-CoV-2), bacterial pathogens (*Mycoplasma pneumoniae*, *Chlamydia pneumoniae*, *Coxiella burnetii*, *Bartonella henselae*, *Brucella* spp., *Toxoplasma gondii*, *Borrelia* spp. with ELISA and Western blot confirmation, *Helicobacter pylori*, and *Yersinia enterocolitica*), and the fungal species *Candida albicans*. Patients with complete results were further classified according to IgM status into those without positive IgM antibodies, those positive for a single microorganism, those positive for two microorganisms, and those positive for three or more microorganisms. For *Borrelia* spp., only Western blot results were considered when calculating the total number of IgM antibodies. In the Lyme disease group, IgM antibodies against *Borrelia* were not included in this overall calculation, as positivity for this pathogen was an inclusion criterion.

### 2.4. Statistical Analysis

Descriptive statistics are presented as counts and percentages, mean ± standard deviation (SD), or median values with interquartile range (IQR, 25th–75th percentile), depending on data type and distribution. Normality was tested using the Smirnov test, complemented by visual inspection of Q–Q plots and detrended Q–Q plots.

Comparisons between groups were performed using analysis of variance (ANOVA) with Tukey’s HSD post hoc test for parametric data, while nonparametric comparisons employed the chi-square test with Bonferroni adjustment for column proportions, as well as the Kruskal–Wallis test with Dunn’s post hoc test and Bonferroni correction. Logistic regression (for binary dependent variables) and multinomial regression (for categorical dependent variables with three or more categories) were applied to assess associations between dependent and independent variables.

A *p*-value < 0.05 was considered statistically significant. Statistical analyses were conducted using SPSS software, version 26.0 (IBM Corp., Armonk, NY, USA).

## 3. Results

Table 1 presents the demographic characteristics of the study population. There were no statistically significant differences between groups in terms of gender distribution or mean age. Patients in the Lyme disease group had a significantly higher proportion of individuals with anamnestic history of syncope. Among the subgroup of patients with syncope, the Lyme disease group also showed significantly higher rates of orthostatic hypotension (OH) during HUTT compared to patients with CFS after COVID-19 and CFS with insidious onset, although not when compared to the PCS group.

Frequencies of different outcomes during HUTT across the study groups are summarized in Table 2. Positive HUTT results were significantly more prevalent in the Lyme disease group compared to the other three groups. In contrast, patients with CFS of insidious onset demonstrated higher rates of small variation in blood pressure (SVBP) during HUTT, with statistical significance observed only when compared to the Lyme disease group.

Partial results of the serological testing are shown in Table 3. The Lyme disease group had a significantly higher median total score of positive IgM antibodies than the other three groups. This group also showed higher rates of IgM positivity for parvovirus B19 when compared to the CFS with insidious onset group, and for coxsackievirus when compared to both CFS after COVID-19 and CFS with insidious onset. Conversely, the prevalence of IgG antibodies against HSV-1 and VZV was significantly higher in all three non-Lyme groups compared with the Lyme disease group.

**Figure 1:**
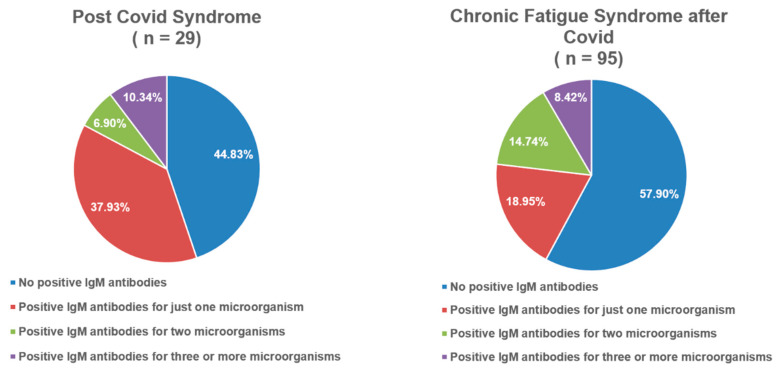
Frequency distribution of IgM-positive antibodies in post-COVID syndrome and chronic fatigue syndrome after COVID-19.

**Figure 2:**
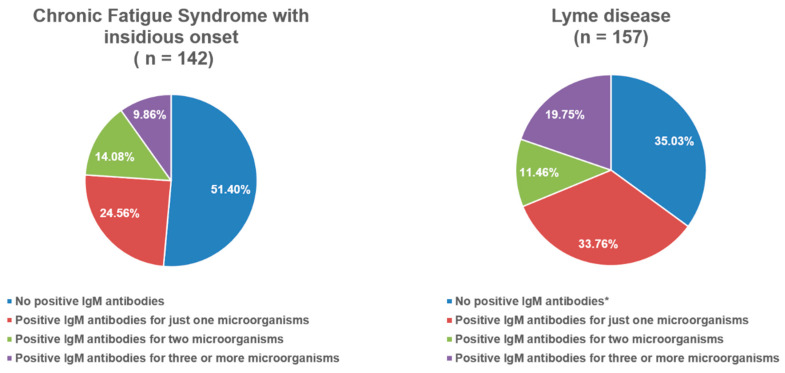
Frequency distribution of IgM-positive antibodies in chronic fatigue syndrome with insidious onset and Lyme disease. *—In Lyme disease group, positive IgM antibodies for *Borrelia* spp. (Elisa and Western blot) were not taken into account when calculating the overall sum of positive IgM antibodies because they were one of the inclusion criteria for that group.

Figure 1 and Figure 2 illustrate the distribution of positive IgM antibodies across the groups. In both the PCS and Lyme disease groups, fewer than half of the patients were negative for IgM antibodies. The proportion of patients with positive IgM antibodies against three or more microorganisms ranged from 8.4% to 19.8%, depending on the group.

Binary logistic regression analyses for different HUTT outcomes are presented in Table 4, Table 5, Table 6 and Table 7. In both CFS subgroups (after COVID-19 and with insidious onset), female gender was identified as a significant positive predictor of positive HUTT in multivariable models. In addition, positivity for IgM antibodies against HSV-1 was a significant predictor in the CFS with insidious onset group. Both models were statistically significant. In the PCS group, hypotension at baseline in the supine position emerged as a significant predictor of positive HUTT in the univariable model. In the Lyme disease group, IgG positivity for Toxoplasma gondii was identified as a positive predictor, again in the univariable model. These models were statistically significant as well.

Hypotension at supine baseline was also a significant positive predictor of OH during HUTT in both the PCS and CFS after COVID-19 groups, with both univariable models reaching statistical significance. In the CFS with insidious onset group, negative IgG antibodies for EBV and positive IgG antibodies for SARS-CoV-2 were significant predictors of OH, while in the Lyme disease group, positive IgM antibodies against *Borrelia* were significant predictors. All corresponding models were statistically significant.

For blood pressure variation outcomes, positive IgG antibodies against adenovirus in the PCS group and male gender in the Lyme disease group were significant predictors of extreme variation in blood pressure (EVBP). Supine SVBP in the PCS group and IgG positivity for *Coxiella burnetii* and SARS-CoV-2 in the CFS after COVID-19 group were significant predictors of SVBP.

Predictors of extreme hypertensive reaction also varied across groups. In the CFS after COVID-19 group, male gender was a significant positive predictor. In the Lyme disease group, positive IgG antibodies against HHV-6 were predictive of extreme hypertensive reaction. Conversely, in the CFS with insidious onset group, positive IgG antibodies against adenovirus were identified as a significant negative predictor. All of these models were statistically significant.

Results of multinomial regression analyses based on serology are presented in Table 8a–c.

Negative IgG antibodies for HSV-1 and VZV were significant positive predictors of group membership in the Lyme disease cohort compared with all other groups. Negative IgG antibodies for SARS-CoV-2 were significant positive predictors of belonging to the CFS with insidious onset and Lyme disease groups when compared with PCS and CFS after COVID-19. Negative IgG antibodies for *Chlamydia pneumoniae* were significant negative predictors for Lyme disease group membership when compared with PCS and CFS with insidious onset. Finally, negative IgM antibody status was identified as a significant positive predictor of Lyme disease group membership when compared with CFS with insidious onset.

## 4. Discussion

With respect to demographic characteristics, Table 1 shows that the highest proportion of patients with a previous history of syncope was in the Lyme disease group, exceeding 50% of participants. Syncope in Lyme disease is most commonly attributed to conduction disturbances, particularly atrioventricular (AV) block, with approximately 15% of patients requiring pacemaker implantation at some point [28,29,30]. However, none of the participants with Lyme disease in the present study exhibited conduction disorders severe enough to necessitate pacemaker placement. Syncope associated with OH during HUTT, as well as OH itself during HUTT, was most frequently observed in the Lyme disease group, although without statistical significance when compared to the other groups (Table 1 and Table 2). Tanking et al. reported abnormal HUTT results in 12 of 15 subjects with PCS, of whom 10 demonstrated neurocardiogenic syncope as the predominant mechanism [31]. In the context of CFS, Kenny and Graham observed that 45–90% of patients experience syncope or light-headedness [32]. Similarly, Bou-Holaigah et al. identified abnormal HUTT responses in 22 of 23 CFS patients, with a significant difference compared to controls [33]. Conversely, Timmers et al. reported postural tachycardia or (pre)syncope in only a minority of CFS patients and attributed these findings largely to deconditioning [34].

In our study population, the prevalence of POTS ranged from 5.4% to 13.7% depending on the group. Prior reports indicate a prevalence between 1.5% and 100% in PCS, influenced by sample size, and consistently demonstrate higher prevalence in PCS compared to CFS of non-COVID origin or following EBV infection [31,35]. Although orthostatic intolerance is often associated with classical OH, delayed OH, or POTS, as noted in the European Society of Cardiology guidelines on syncope [27], approximately half of the patients in all four groups demonstrated extreme blood pressure variations during HUTT, potentially impairing cerebral perfusion. Hausenloy et al. reported that blood pressure variability during HUTT had an 87% positive predictive value for vasovagal syncope [36]. Nonetheless, other studies have identified reduced cerebral blood flow in CFS patients, even in the absence of hemodynamic changes, suggesting that cerebral vascular control is closely linked to skeletal muscle pH [37,38,39].

Regarding serological findings, Figure 1 and Figure 2 indicate that between 55% and 65% of patients in the PCS and Lyme disease groups were positive for at least one IgM antibody, while more than 10% demonstrated positivity for three or more microorganisms. Prior studies of PCS have highlighted EBV reactivation during acute COVID-19, with implications as a marker of disease severity [40]. Milovanović et al. described severe autonomic dysfunction during the first two weeks of acute COVID-19 [41], while Vojdani et al. suggested that the persistence of SARS-CoV-2, as well as the reactivation of latent viruses such as EBV and HHV-6, may contribute to PCS pathophysiology [42]. EBV, HHV-6, SARS-CoV-2, and other microorganisms including *Coxiella burnetii* are also recognized contributors to the development of CFS [43,44]. In our subgroup of patients with CFS of insidious onset, 48.2% were positive for at least one IgM antibody (Figure 2). The Lyme disease group demonstrated higher rates of IgM positivity for parvovirus B19 and adenovirus, with statistically significant differences compared to some groups (Table 3). Parvovirus B19 is well established as a co-infection in Lyme disease and is particularly relevant in differential diagnosis due to its association with persistent arthropathy and myocarditis [22]. Perlejewski et al. further identified adenovirus, HSV-1, HSV-2, EBV, CMV, and other enteroviruses in 13% of patients with Lyme neuroborreliosis [45]. In our study, fewer than 40% of Lyme disease patients were positive for IgG against HSV-1 or VZV, which was significantly lower than in the other groups. Statistically significant differences were also observed for EBV and CMV IgG positivity compared with certain groups (Table 3). Previous reports have described the reactivation of latent Lyme disease during acute HSV-1 infection and EBV/HSV-1 co-infections during acute Lyme disease [46,47,48].

Binary logistic regression provided additional insights. In the PCS group, IgG positivity for adenovirus emerged as a significant negative predictor of blood pressure variability (Table 4). In the CFS with insidious onset group, adenovirus IgG was also a significant negative predictor of hypertensive reactions (Table 6). Hannestad et al. reported significantly elevated salivary IgG antibodies to adenovirus in patients with ME/CFS after COVID-19 compared to healthy donors, suggesting that impaired antiviral immunity may facilitate adenovirus reactivation [49]. In contrast, IgM positivity for adenovirus was a significant positive predictor of OH in the Lyme disease group (Table 7). Although no direct relationship between adenovirus and autonomic dysfunction has been established, positive IgM for *Chlamydia pneumoniae* was also identified as a significant predictor of POTS in the Lyme group. While C. pneumoniae has not been directly linked to POTS, Chémali and Sandefer reported associations between highly elevated *Mycoplasma pneumoniae* titers, autoimmune disorders, and POTS or small fiber neuropathy [50]. Moreover, IgG positivity for Toxoplasma gondii and HHV-6 in the Lyme disease group were predictors of positive HUTT outcomes and extreme hypertensive reactions, suggesting that co-infections or prior infections may contribute to dysautonomia in this population.

In CFS with insidious onset, in addition to female gender, IgM positivity for HSV-1 was a significant predictor of positive HUTT outcomes (Table 6). HSV-1 has previously been shown to establish latency in autonomic ganglia and propagate along axonal pathways [51,52]. In CFS after COVID-19, IgG positivity for *Coxiella burnetii* and SARS-CoV-2 were significant predictors of SVBP during HUTT, consistent with proposed mechanisms of ANS injury in COVID-19 and the observation that approximately 12% of patients with acute *Coxiella* infection develop CFS [18]. These findings suggest that post-COVID-19 patients with prior *Coxiella* infection may experience more severe ANS dysfunction.

Multinomial regression also revealed notable findings. In addition to expected associations with *Borrelia* spp. and SARS-CoV-2 antibodies, IgM positivity for *Bartonella* emerged as a significant predictor of CFS group membership rather than Lyme disease (Table 8c). Although *Bartonella* is considered a common co-infection in Lyme disease, these results raise the possibility that unrecognized *Bartonella* infection may also contribute to the development of CFS [22].

### Study Limitations

This study has several limitations. First, the number of patients differed considerably across groups, which may have affected statistical power and the comparability of results. Second, not all patients in the PCS group or in the CFS groups (post-COVID-19 and insidious onset) had complete serological data, limiting the inclusion of these cases in statistical analyses.

## 5. Conclusions

In patients with post-infectious syndromes, including post-COVID syndrome, chronic fatigue syndrome, and late-stage Lyme disease, damage to the autonomic nervous system manifests through a spectrum of hemodynamic abnormalities, ranging from extreme blood pressure variability to postural orthostatic tachycardia syndrome (POTS) and orthostatic hypotension. Head-up tilt testing was most frequently positive in patients with late-stage Lyme disease. More than half of the patients with post-COVID syndrome and late-stage Lyme disease exhibited IgM positivity for at least one microorganism.

Binary logistic regression analysis demonstrated that, in addition to classical hemodynamic parameters, serological evidence of infection, particularly positive antibodies against specific microorganisms, constituted significant predictors of hemodynamic responses during HUTT. Furthermore, multinomial regression analysis indicated that negative IgG antibody status was a significant predictor of classification into the late-stage Lyme disease group rather than the other post-infectious groups.

Taken together, these findings suggest that autonomic nervous system dysfunction in post-infectious syndromes may be, at least in part, a consequence of persistent or prior infections.

## Figures and Tables

**Table 1:** Demographic characteristics of the study population.

	PCS (n = 95)	CFS After Cov. (n = 285)	CFS (n = 499)	Lyme (n = 157)	*p*
Male (n, %)	37 (39.95%)	87 (30.53%)	157 (31.46%)	53 (33.76%)	0.45 ^1^
Female (n, %)	60 (60.05%)	195 (69.47%)	342 (68.64%)	104 (66.24%)	
Age (mean + SD)	45.88 ± 13.60	46.26 ± 13.30	44.31 ± 12.79	44.76 ± 14.92	0.231 ^2^
History of Syncope (n, %)	30 (31.60%)	117 (41.10%)	176 (35.30%)	64 (57.10%) ^+; #;^ *	<0.001 ^1^
+Syncop. and −OH (n, %)	19 (20.00%)	78 (27.40%)	109 (21.9%)	35 (31.3%)	0.072 ^1^
+Syncop. and +OH (n, %)	11 (11.60%)	39 (13.70%)	67 (13.40%)	29 (25.90%) ^#;^ *	0.005 ^1^
−Syncop. And +OH (n, %)	4 (4.20%)	18 (6.30%)	28 (5.60%)	4 (3.60%)	0.685 ^1^

PCS—post-COVID syndrome; CFS after Cov.—chronic fatigue syndrome after COVID-19; CFS—chronic fatigue syndrome with insidious onset; +Syncop. and –OH; had syncope before and did not have orthostatic hypotension during head-up tilt test; +Syncope. and +OH—had syncope before and had orthostatic hypotension during head-up tilt test; −Syncope. And +OH—no history of syncope and had orthostatic hypotension during head-up tilt test; ^1^—Pearson Chi-Square, with column proportion comparison using Bonferroni method; +—*p* < 0.05, after Bonferroni correction; for comparing column proportion of PCS group with other three groups, respectively; #—*p* < 0.05, after Bonferroni correction; for comparing column proportion of CFS after Cov. group with CFS group and Lyme group, respectively; *—*p* < 0.05, after Bonferroni correction; for comparing column proportion of CFS group with Lyme group. ^2^—One-Way ANOVA.

**Table 2:** Outcomes during head-up tilt test between groups.

	PCS (n = 95)	CFS After Cov. (n = 285)	CFS (n = 499)	Lyme (n = 112)	*p*
Positive HUTT (n, %)	26 (27.40%)	96 (33.70%)	145 (29.10%)	56 (50%) ^+; #;^ *	<0.001 ^1^
EVBP during HUTT (n, %)	44 (46.30%)	155 (54.40%)	257 (51.50%)	52 (46.40%)	0.531 ^1^
SMVP during HUTT (n, %)	9 (9.50%)	36 (12.60%)	98 (19.60%)	11 (9.80%) *	0.001 ^1^
OH during HUTT (n, %)	15 (15.8%)	57 (20.00%)	95 (19.00%)	33 (29.50%)	0.162 ^1^
POTS during HUTT (n, %)	13 (13.70%)	24 (8.40%)	32 (6.40%)	6 (5.40%)	0.102 ^1^
Hypertens. reaction (n, %)	18 (18.90%)	57 (20.00%)	119 (23.80%)	17 (15.20%)	0.259 ^1^
Extrem. Hypertens. reaction (n, %)	7 (7.40%)	34 (11.90%)	50 (10.00%)	8 (7.10%)	0.322 ^1^
Normal response (n, %)	25 (26.30%)	42 (14.70%)	87 (17.40%)	13 (11.60%) ^+^	0.026 ^1^

PCS—post-COVID syndrome; CFS after Cov.—chronic fatigue syndrome after COVID-19; CFS—chronic fatigue syndrome with insidious onset; HUTT—head-up tilt test; EVBP—extreme blood pressure variations during HUTT; SVBP—small blood pressure variations during HUTT; OH—orthostatic hypotension during HUTT; POTS—postural orthostatic tachycardia syndrome during HUTT; Hypertens.—hypertensive; Extrem. Hypertens.—extreme hypertensive; ^1^—Pearson Chi-Square, with column proportion comparison using Bonferroni method; +—*p* < 0.05, after Bonferroni correction; for comparing column proportion of PCS group with other three groups, respectively; #—*p* < 0.05, after Bonferroni correction; for comparing column proportion of CFS after Cov. group with CFS and Lyme group, respectively; *—*p* < 0.05, after Bonferroni correction; for comparing column proportion of the CFS group with Lyme group.

**Table 3:** Results of serology tests, where there is statistical significance, among the groups.

	PCS (n = 29)	CFS After Cov. (n = 95)	CFS (n = 142)	Lyme (n = 157)	*p*
Total score of pos. IgM (Mdn + IQR)	1 (0–1)	0 (0–1)	0 (0–1)	1 (0–2) ^”; &^	0.003 ^1^
**Percentage of positive IgM antibodies among the groups**					
Pos. IgM for Adenovirus	0 (0%)	5 (5.30%)	13 (9.20%)	21 (13.40%)	0.047 ^2^
Pos. IgM for Parvo B19	1 (3.40%)	2 (2.10%)	2 (1.40%)	14 (8.90%) *	0.009 ^2^
Pos. IgM for Coxsackiae	1 (3.40%)	3 (3.20%)	5 (3.50%)	35 (22.30%) ^#;^ *	<0.001 ^2^
**Percentage of positive IgG antibodies among the groups**					
Pos. IgG for Adenovirus	16 (55.20%)	68 (71.60%)	79 (55.60%)	57 (36.30%) ^#;^ *	<0.001 ^2^
Pos. IgG for Coxsackiae	4 (13.80%)	11 (11.60%)	9 (6.30%)	29 (18.50%) *	0.017 ^2^
Pos. IgG for CMV	25 (86.20%)	70 (73.70%)	107 (75.40%)	92 (58.60%) ^+;^ *	0.001 ^2^
Pos. IgG for EBV	24 (82.80%)	77 (81.10%)	106 (74.60%)	93 (59.20%) ^#;^ *	<0.001 ^2^
Pos. IgG for Candida	1 (3.40%)	5 (5.30%)	18 (12.70%)	6 (3.80%) *	0.016 ^2^
Pos. IgG for VZV	25 (86.20%)	77 (81.10%)	112 (78.90%)	60 (38.20%) ^+; #;^ *	<0.001 ^2^
Pos. IgG for HSV1	21 (72.40%)	63 (66.30%)	93 (63.50%)	40 (25.50%) ^+; #;^ *	<0.001 ^2^
Pos. IgG for HSV2	3 (10.30%)	14 (14.70%)	23 (16.20%)	9 (5.70%) *	0.027 ^2^

PCS—post-COVID syndrome; CFS after Cov.—chronic fatigue syndrome after COVID-19; CFS—chronic fatigue syndrome with insidious onset; pos.—positive; Mdn—median; IQR—interquartile range (25–75%); CMV—cytomegalovirus; EBV—Ebstein Barr Virus; VZV—Varicella Zooster Virus; HSV1—Herpes Simplex Virus 1; HSV2—Herpes Simplex Virus 2; ^1^—Kruskal–Wallis non-parametric test followed by Dunn’s test with Bonferroni correction. ^”^—*p* = 0.005, when comparing the CFS after Cov. group with Lyme group. ^&^—*p* = 0.028, when comparing the CFS after Cov. group with Lyme group. ^2^—Pearson Chi-Square, with column proportion comparison using Bonferroni method; +—*p* < 0.05, after Bonferroni correction; for comparing column proportion of PCS group with other three groups, respectively; #—*p* < 0.05, after Bonferroni correction; for comparing column proportion of CFS after Cov. group with CFS and Lyme group, respectively; *—*p* < 0.05, after Bonferroni correction; for comparing column proportion of the CFS group with Lyme group.

**Table 4:** Predicting outcomes during head-up tilt test in patients with post-COVID syndrome.

Variable	Univariable Model	
	Exp (B) (95% CI)	*p*
**Positive head-up tilt test ^a^**		
Hypotension at supine	6.09 (1.04–35.56)	0.045
**EVBP during head-up tilt test ^b^**		
Pos. IgG for Adenovirus	0.15 (0.03–0.76)	0.022
**SVBP during head-up tilt test ^c^**		
SVBP at supine	12.00 (1.46–98.60)	0.021
**OH ^d^**		
Hypotension at supine	14.18 (2.32–86.74)	0.008
**Hypertensive reaction (>130/90 mmHg) ^e^**		
Pos. IgA for Helicobacter Pylori	34.5 (2.35–505.75)	0.01

EVBP—extreme variations of blood pressure; Pos.—positive; SVBP—small variations of blood pressure; orthostatic hypotension. ^a^ Univariable model for positive head-up tilt test: Pearson Chi-Square: *χ*^2^ = 4.328, *p* = 0.037; R^2^ = 0.064. ^b^ Univariable model for extreme blood pressure variation: Pearson Ch- Square: *χ*^2^ = 5.849, *p* = 0.016; R^2^ = 0.244. ^c^ Univariable model for small blood pressure variation: Pearson Chi-Square: *χ*^2^ = 4.637, *p* = 0.031; R^2^ = 0.102. ^d^ Univariable model for orthostatic hypotension: Pearson Chi-Square: *χ*^2^ = 8.656, *p* = 0.002; R^2^ = 0.150. ^e^ Univariable model for hypertensive reaction: Pearson Chi-Square: *χ*^2^ = 8.225, *p* = 0.004; R^2^ = 0.411.

**Table 5:** Predicting outcomes during head-up tilt test in patients with CFS after COVID-19.

Variable	Univariable		Multivariable	
	Exp (B) (95% CI)	*p*	Exp (B) (95% CI)	*p*
	**Positive head-up tilt test ^a^**			
Female	2.95 (1.60–5.44)	0.001	10.03 (2.52–39.95)	0.001
SVBP at Supine	0.23 (0.07–0.77)	0.017		
Hypotension at Supine	6.34 (1.25–32.02)	0.025		
Pos. IgG for Adenovirus	2.86 (1.07–7.64)	0.036		
Pos. IgA for Helicobacter	10.91 (1.29–92.66)	0.029		
Pos. IgG for *Bartonella*	9.09 (1.05–78.75)	0.045		
	**SVBP pressure during head-up tilt test ^b^**			
Pos. IgG for *Coxiella burnetii*	12.15 (1.03–143.85)	0.048	21.00 (1.62–264.33)	0.019
Pos. IgG for SARS-CoV-2	3.50 (1.11–11.09)	0.033	4.59 (1.36–15.57)	0.014
	**OH ^c^**			
Hypotension at Supine	7.21 (1.67–31.14)	0.008		
	**Extreme hypertensive reaction (>170/120 mmHg) ^d^**			
Male	3.41 (1.64–7.09)	0.001	17.86 (2.03–166.67)	0.009
Tachycardia at Supine	5.98 (1.278–27.95)	0.023		
Pos. IgG for Adenovirus	0.20 (0.05–0.92)	0.039		

CFS—chronic fatigue syndrome; SVBP—small variations of blood pressure; Pos.—positive. ^a^ Multivariable model for positive head-up tilt test: Pearson Chi-Square: *χ*^2^ = 38.850. *p* < 0.001; R^2^ = 0.448. ^b^ Multivariable model for small blood pressure variation: Pearson Chi-Square: *χ*^2^ = 10.014. *p* = 0.007; R^2^ = 0.172. ^c^ Univariable model for orthostatic hypotension: Pearson Chi-Square: *χ*^2^ = 7.113. *p* = 0.008; R^2^ = 0.039. ^d^ Multivariable model for extreme hypertensive reaction: Pearson Chi-Square: *χ*^2^ = 15.169, *p* = 0.002; R^2^ = 0.336.

**Table 6:** Predicting outcomes during head-up tilt test in patients with CFS with insidious onset.

Variable	Univariable		Multivariable	
	Exp (B) (95% CI)	*p*	Exp (B) (95% CI)	*p*
	**Positive head-up tilt test ^a^**			
Female	3.45 (2.09–5.69)	<0.001	7.28 (2.50–21.18)	<0.001
Pos. IgM for HSV1	4.93 (1.25–19.48)	0.023	6.51 (1.40–30.36)	0.017
	**SVBP during head-up tilt test ^b^**			
Male	1.58 (1.01–2.51)	0.049		
	**POTS ^c^**			
Pos. IgM for Candida	15.00 (1.19–189.42)	0.036		
	**Orthostatic hypotension ^d^**			
Hypotension at Supine	5.56 (1.46–21.10)	0.012		
Female	2.08 (1.21–3.57)	0.008		
Pos. IgG antibodies for EBV	0.37 (0.16–0.84)	0.017	0.30 (0.12–0.73)	0.008
Pos. IgG for SARS-CoV-2	3.74 (1.21–11.59)	0.022	3.90 (1.15–13.21)	0.029
	**Hypertensive reaction (>130/90 mmHg) ^e^**			
EVBP during Supine	6.57 (1.19–36.35)	0.031		
Pos. IgG for Adenovirus	0.28 (0.11–0.70)	0.007	0.29 (0.11–0.77)	0.008
Pos. IgG for Helicobacter	4.04 (1.01–16.24)	0.049		
	**Extreme hypertensive reaction (>170/120 mmHg) ^f^**			
Male	2.00 (1.11–3.62)	0.021		
HTA at Supine	6.19 (1.01–38.00)	0.049		
Pos. IgG for Adenovirus	0.16 (0.03–0.75)	0.020	0.138 (0.03–0.74)	0.021
Pos. IgG for Helicobacter	7.81 (1.64–37.16)	0.010		
Pos. IgG for Toxoplasma	5.67 (1.45–22.18)	0.013		

CFS—chronic fatigue syndrome; HSV1—Herpes Simplex Virus 1; Pos.—positive; EBV—Epstein–Barr Virus; EVBP—extreme variation of blood pressure; HTA—hypertension; POTS—postural orthostatic tachycardia syndrome; SVBP—small variations of blood pressure. ^a^ Multivariable model for positive head-up tilt test: Pearson Chi-Square: *χ*^2^ = 23.998, *p* < 0.001; R^2^ = 0.212. ^b^ Univariable model for small variation of blood pressure: Pearson Chi-Square: *χ*^2^ = 3.810, *p* = 0.051; R^2^ = 0.012. ^c^ Univariable model for POTS: Pearson Chi-Square: *χ*^2^ = 3.038, *p* = 0.081; R^2^ = 0.094. ^d^ Multivariable model for orthostatic hypotension: Pearson Chi-Square: *χ*^2^ = 18.771, *p* = 0.001; R^2^ = 0.186. ^e^ Multivariable model for hypertensive reaction: Pearson Chi-Square: *χ*^2^ = 11.104, *p* = 0.04; R^2^ = 0.122. ^f^ Multivariable model for extreme hypertensive reaction: Pearson Chi-Square: *χ*^2^ = 15.861, *p* = 0.007; R^2^ = 0.252.

**Table 7:** Predicting outcomes during head-up tilt test in patients with Lyme disease.

Variable	Univariable		Multivariable	
	Exp (B) (95% CI)	*p*	Exp (B) (95% CI)	*p*
	**Positive head-up tilt test ^a^**			
Pos. IgG for Toxoplasma	11.00 (1.34–90.62)	0.026		
	**EVBP during head-up tilt test ^b^**			
Male	3.82 (1.63–8.93)	0.002		
	**POTS ^c^**			
Pos. IgM for Chlamydia Pneumoniae	34.00 (1.52–760.85)	0.26		
	**Orthostatic hypotension ^d^**			
Pos. IgM for Adenovirus	4.62 (1.36–15.72)	0.014		
	**Extreme hypertensive reaction (>170/120) ^e^**			
Pos. IgM for Candida Albicans	22.33 (1.11–450.13)	0.043		
Pos. IgG for Candida Albicans	33.00 (2.96–368.36)	0.005		
Pos. IgG for HHV6	12.60 (1.45–109.39)	0.022	19.72 (1.04–373.10)	0.047
Pos. IgG for SARS-CoV-2	12.60 (1.45–109.39)	0.022		

Pos.—positive; EVBP—extreme variations of blood pressure; POTS—postural orthostatic tachycardia syndrome; HHV6—Human Herpes Virus 6. ^a^ Univariable model for Positive head-up tilt test: Pearson Chi-Square: *χ*^2^ = 8.358, *p* = 0.004; R^2^ = 0.147. ^b^ Univariable model for extreme blood pressure during head-up tilt test: Pearson Chi-Square: *χ*^2^ = 10.175, *p* = 0.001; R^2^ = 0.116. ^c^ Univariable model for POTS during head-up tilt test: Pearson Chi-Square: *χ*^2^ = 4.005, *p* = 0.045; R^2^ = 0.185. ^d^ Univariable model for orthostatic hypotension: Pearson Chi-Square: *χ*^2^ = 6.079, *p* = 0.014; R^2^ = 0.116. ^e^ Multivariable model for extreme hypertensive reaction: Pearson Chi-Square: *χ*^2^ = 11.577, *p* = 0.043; R^2^ = 0.426.

**Table 8:** (**a**). Multinomial regression for groups based on results of serology testing. (**b**). Multinomial regression for groups based on results of serology testing. (**c**). Multinomial regression for groups based on results of serology testing.

(a) Reference Category: Post COVID Syndrome *					
		Parameter Estimate			
Group	Variable	*p*	Exp (B)	95% CI	
**CFS after COVID-19**	Neg. IgG for Adenovirus ^a^	0.017	0.22	0.07–0.77	
	Pos. IgG for Adenovirus	0 *			
**CFS with insidious onset**	Neg. IgG for Coxsackiae virus ^b^	0.013	8.35	1.58–44.25	
	Pos. IgG for Coxsackiae virus	0 *			
	Neg. IgG for SARS-CoV-2 ^c^	0.026	6.00	1.24–28.97	
	Pos. IgG for SARS-CoV-2	0 *			
**Lyme disease**	Neg. WB IgM for *Borrelia* spp. ^d^	<0.001	0.001	0.01–0.08	
	Pos. WB IgM for *Borrelia* spp.	0 *			
	Neg. IgG for Chlamydia Pneumoniae ^e^	0.044	0.23	0.06–0.96	
	Pos. IgG for Chlamydia Pneumoniae	0 *			
	Neg. IgG for VZV ^f^	0.018	9.97	1.48–67.40	
	Pos. IgG for VZV	0 *			
	Neg. IgG for HSV1 ^g^	<0.001	21.70	3.91–120.57	
	Pos. IgG for HSV1	0 *			
	Neg. IgG for SARS-CoV-2	0.029	13.15	1.31–131.91	
	Pos. IgG for SARS-CoV-2	0 *			
(b) Reference Category: CFS After COVID-19 *					
		**Parameter Estimate**			
**Group**	**Variable**	***p***	**Exp (B)**	**95% CI**	
**CFS with insidious onset**	Neg. IgG for Chlamydia Pneumoniae ^a^	0.030	2.08	1.07–4.01	
	Pos. IgG for Chlamydia Pneumoniae	0 *			
	Neg. IgG for Candida Albicans ^b^	0.048	0.29	0.08–0.99	
	Pos. IgG for Candida Albicans	0 *			
	Neg. IgG for SARS-CoV-2 ^c^	0.007	4.03	1.45–11.17	
	Pos. IgG for SARS-CoV-2	0 *			
**Lyme disease**	Neg. Elisa IgM for *Borrelia* spp. ^d^	<0.001	0.05	0.01–0.21	
	Pos. Elisa IgM for *Borrelia* spp.	0 *			
	Neg. Elisa IgG for *Borrelia* spp. ^e^	0.005	0.05	0.01–0.41	
	Pos. Elisa IgG for *Borrelia* spp.	0 *			
	Neg. WB IgM for *Borrelia* spp. ^f^	<0.001	0.01	0.01–0.06	
	Pos. WB IgM for *Borrelia* spp.	0 *			
	Neg. WB IgG for *Borrelia* spp. ^g^	<0.001	0.015	0.01–0.08	
	Pos. WB IgG for *Borrelia* spp.	0 *			
	Neg. IgG for VZV ^h^	0.02	5.23	1.30–21.08	
	Pos. IgG for VZV	0 *			
	Neg. IgG for HSV1 ^i^	0.004	6.93	1.85–25.97	
	Pos. IgG for HSV1	0 *			
	Neg. IgG for SARS-CoV-2	0.026	8.84	1.30–60.02	
	Pos. IgG for SARS-CoV-2	0 *			
(c) Reference Category: CFS with Insidious Onset *					
		**Parameter Estimates**			
**Group**	**Variables**	***p***	**Exp (B)**	**95% CI**	
**Lyme disease**	Neg. Elisa IgM for *Borrelia* spp. ^a^	<0.001	0.03	0.01–0.13	
	Pos. Elisa IgM for *Borrelia* spp.	0 *			
	Neg. Elisa IgG for *Borrelia* spp. ^b^	0.003	0.05	0.01–0.36	
	Pos. Elisa IgG for *Borrelia* spp.	0 *			
	Neg. WB IgM for *Borrelia* spp. ^c^	<0.001	0.01	0.01–0.06	
	Pos. WB IgM for *Borrelia* spp.	0 *			
	Neg. WB IgG for *Borrelia* spp. ^d^	<0.001	0.01	0.01–0.03	
	Pos. WB IgG for *Borrelia* spp.	0 *			
	Neg. IgM for Coxsackiae virus ^e^	0.013	0.06	0.01–0.56	
	Pos. IgM for Coxsackiae virus	0 *			
	Neg. IgG for Chlamydia Pneumoniae ^f^	0.020	0.25	0.08–0.80	
	Pos. IgG for Chlamydia Pneumoniae	0 *			
	Negative IgG for VZV ^g^	0.001	9.28	2.39–36.12	
	Pos. IgG for VZV	0 *			
	Negative IgG for HSV1 ^h^	0.006	6.07	1.66–22.17	
	Pos. IgG for HSV1	0 *			
	Neg. IgM for *Bartonella* Henselae ^i^	0.048	17.59	1.02–302.39	
	Pos. IgM for *Bartonella* Henselae	0 *			

(**a**) *—R^2^ = 0.755; 0 *.—the parameter is set to zero because its redundant; CFS—chronic fatigue syndrome; Neg.—negative; Pos.—positive; WB—Western blot; VZV—Varicella Zoster Virus; HSV1—Herpes Simplex Virus 1. Likelihood ratio tests: ^a^ Pearson Chi-Square: *χ*^2^ = 6.110, *p* = 0.106; ^b^ Pearson Chi-Square: *χ*^2^ = 7.944, *p* = 0.047; ^c^ Pearson Chi-Square: *χ*^2^ = 13.198, *p* = 0.004; ^d^ Pearson Chi-Square: *χ*^2^ = 66.891, *p* < 0.001; ^e^ Pearson Chi-Square: *χ*^2^ = 9.34, *p* = 0.025; ^f^ Pearson Chi-Square: *χ*^2^ = 11.886, *p* = 0.008; ^g^ Pearson Chi-Square: *χ*^2^ = 14.642, *p* = 0.002. (**b**) *—R^2^ = 0.755; 0 *.—the parameter is set to zero because its redundant; CFS—chronic fatigue syndrome; Neg.—negative; Pos.—positive; WB—Western blot; VZV—Varicella Zoster Virus; HSV1—Herpes Simplex Virus 1. Likelihood ratio tests: ^a^ Pearson Chi-Square: *χ*^2^ = 9.354, *p* = 0.025; ^b^ Pearson Chi-Square: *χ*^2^ = 5.263, *p* = 0.154; ^c^ Pearson Chi-Square: *χ*^2^ = 13.198, *p* = 0.004; ^d^ Pearson Chi-Square: *χ*^2^ = 37.613, *p* < 0.001; ^e^ Pearson Chi-Square: *χ*^2^ = 15.124, *p* = 0.002; ^f^ Pearson Chi-Square: *χ*^2^ = 71.288, *p*< 0.001; ^g^ Pearson Chi-Square: *χ*^2^ = 66.891, *p*< 0.001; ^h^ Pearson Chi-Square: *χ*^2^ = 11.886, *p* = 0.008; ^i^ Pearson Chi-Square: *χ*^2^ = 14.642, *p* = 0.002. (**c**) *—R^2^ = 0.755; 0 *.—the parameter is set to zero because its redundant; chronic fatigue syndrome; Neg.—negative; Pos.—positive; WB—Western blot; VZV—Varicella Zoster Virus; HSV1—Herpes Simplex Virus 1. Likelihood ratio tests: ^a^ Pearson Chi-Square: *χ*^2^ = 37.613, *p* < 0.001; ^b^ Pearson Chi-Square: *χ*^2^ = 15.124, *p* = 0.002; ^c^ Pearson Chi-Square: *χ*^2^ = 71.288, *p* < 0.001; ^d^ Pearson Chi-Square: *χ*^2^ = 66.891, *p* < 0.001; ^e^ Pearson Chi-Square: *χ*^2^ = 7.944, *p* = 0.047; ^f^ Pearson Chi-Square: *χ*^2^ = 9.354, *p* = 0.025; ^g^ Pearson Chi-Square: *χ*^2^ = 11.886, *p* = 0.008; ^h^ Pearson Chi-Square: *χ*^2^ = 14.642, *p* = 0.002; ^i^ Pearson Chi-Square: *χ*^2^ = 5.217, *p* = 0.157.

## Data Availability

Available upon reasonable request due to privacy reasons.

## Abbreviations

QASAT	Quantitative Scale for Grading of Cardiovascular Autonomic Reflex Tests and Small Fibers from Skin Biopsies
COMPASS-31	Composite Autonomic Symptom Score
SAS	Survey of Autonomic Symptoms
POTS	Postural Orthostatic Tachycardia Syndrome
ESC	European Society of Cardiology
